# Correlation of Computed Tomography Test Bolus Dynamics and Conventional Computed Tomography Parameters With Pulmonary Vascular Resistance in Patients With Pulmonary Arterial Hypertension

**DOI:** 10.7759/cureus.13577

**Published:** 2021-02-26

**Authors:** Dhiraj Baruah, Sushilkumar Sonavane, Lawrence Goodman, Hrudaya Nath, Kenneth Presberg, Kaushik Shahir

**Affiliations:** 1 Radiodiagnosis, Medical University of South Carolina, Charleston, USA; 2 Radiology, Mayo Clinic, Jacksonville, USA; 3 Radiology, Medical College of Wisconsin, Milwaukee, USA; 4 Radiology, University of Alabama, Birmingham, USA; 5 Pulmonary Medicine, Medical College of Wisconsin, Milwaukee, USA; 6 Radiology, Aurora Health Care, Milwaukee, USA

**Keywords:** pulmonary arterial hypertension, computed tomography angiography, cardiac catheterization, pulmonary vascular resistance

## Abstract

Objective: Pulmonary vascular resistance (PVR) is a measurement obtained with invasive right heart catheterization (RHC) that is commonly used for management of patients with pulmonary arterial hypertension (PAH). Computed tomography pulmonary angiography (CTPA) is also done as part of the workup for PAH in some cases. The aim of our study was to assess the correlation of contrast dynamic changes in the main pulmonary artery (MPA) on CTPA with PVR obtained with RHC.

Methods: This is an IRB-approved retrospective study performed in two separate institutions (Medical College of Wisconsin and University of Alabama) between January 2010 and December 2013. During CTPA done as test bolus, serial images are acquired at the level of MPA after intravenous injection of contrast to determine timing of the CT acquisition. Since the PVR changes with the degree of PAH, we hypothesize that will be reflected in the contrast kinetics in MPA. A correlation of standard CT metrics (MPA diameter, right pulmonary artery [PA] diameter, left PA diameter, MPA/aorta ratio, and right ventricle/left ventricle [RV/LV] ratio) and dynamic (full width at half maximum) CTPA parameters in patients with known PAH was performed with PVR obtained from RHC done within 30 days. Statistical analysis was performed by Pearson correlation coefficient.

Results: Among 221 patients in our database, 37 patients fulfilled the selection criteria. There was a strong correlation between full width half maximum (FWHM) and mean pulmonary artery pressure (mPAP) (r=0.69, p value<0.00001), PVR (r=0.8, p value<0.00001) and indexed PVR (PVRI) (r=0.75, p value<0.00001).

Conclusion: FWHM obtained from CTPA strongly correlates with RHC parameters and is potentially more helpful than static measurements for follow-up of patients with known PAH to assess response to treatment or progression.

## Introduction

Pulmonary arterial hypertension (PAH) is defined as a mean pulmonary artery pressure (mPAP) of 25 mm Hg or more at rest, measured at right heart catheterization (RHC) [1]. Pulmonary vascular resistance (PVR) is an important parameter not only in determining severity and degree of pulmonary hypertension but also in the management of PAH and serves as a primary endpoint to assess the treatment efficacy in several newer targeted therapies [2]. Currently, invasive RHC is the gold standard to accurately assess the severity of pulmonary hypertension by providing hemodynamic measures such as PVR and mPAP. Recent work has demonstrated a strong correlation between contrast bolus kinetics measured in the main pulmonary artery from magnetic resonance (MR) angiography and the measurements from RHC in PAH [3,4]. There are imaging parameters on computed tomography (CT) that have shown a good correlation with mPAP, such as size of the main pulmonary artery (PA), right main PA, left main PA, right/left ventricular ratio and PA/aorta ratio [5-7]. However, currently there are no established CT measurements that correlate with PVR. A test bolus is routinely performed prior to CT pulmonary angiography as a planning tool to determine the timing required for the optimum opacification of the pulmonary arteries where a series of images are obtained at the level of the main pulmonary artery after intravenous injection of a small volume of contrast (15-20 ml) via peripheral venous access. This dynamic data has an excellent potential to evaluate pulmonary circulation hemodynamics. We propose an innovative method using test bolus imaging parameters performed for routine computed tomography pulmonary angiography (CTPA) to correlate with PVR.

## Materials and methods

This is an institutional review board (IRB)-approved retrospective study performed in two separate institutions (Medical College of Wisconsin and University of Alabama) and adhered to Health Insurance Portability and Accountability Act requirements. Patient’s consent was waived as per IRB guidelines.

Patients with known PAH (of all types described in the World Health Organization classification [7]) who had a CTPA study (between January 2010 to December 2013) within one month of RHC were included in the study. The exclusion criteria for test bolus dynamics assessment included inadequate scan duration of the test bolus (bolus without a peak and at least part of the descending curve), motion artifacts, and streak artifacts limiting placement of accurate region of interest (ROI) for density measurement. Further, patients with aortic ectasia/aneurysm (ascending aorta more than 4 cm) were excluded from the main pulmonary artery/aorta ratio measurement.

CTPA technique

CTPA was performed on a 64 slice multidetector CT (Discovery 750 HD & VCT; GE, Waukesha, WI, USA) in both the institutions. Similar protocols were used for bolus tracking in both institutions injecting 15 mL of intravenous nonionic contrast (Omnipaque 350; GE) at 3 ml/sec via an upper extremity venous access and repeated axial images with breath hold at the level of the main pulmonary artery, using 100 - 140 kV and 250-450 mA and 2.5 mm slice thickness, scanned at two-second intervals until the contrast opacification of the ascending aorta.

Image analysis

CTPA images were analyzed on dedicated picture archiving and communication system (PACS) workstations (Mckesson, Philips iSite) by fellowship-trained board-certified cardiothoracic radiologists with 10 and 12 years of experience in one institute and eight and 25 years of experience in the other institute. The radiologists were blinded to the RHC data. ROI (size = 1 cm2) was placed on the center of the main pulmonary artery (MPA) and a time attenuation curve was generated based on attenuation values in Hounsfield Units (HU). From this time attenuation curve, full width at half maximum (FWHM) was calculated. FWHM of the test bolus is the width of the main pulmonary artery enhancement curve at half its maximum density.

**Figure 1:**
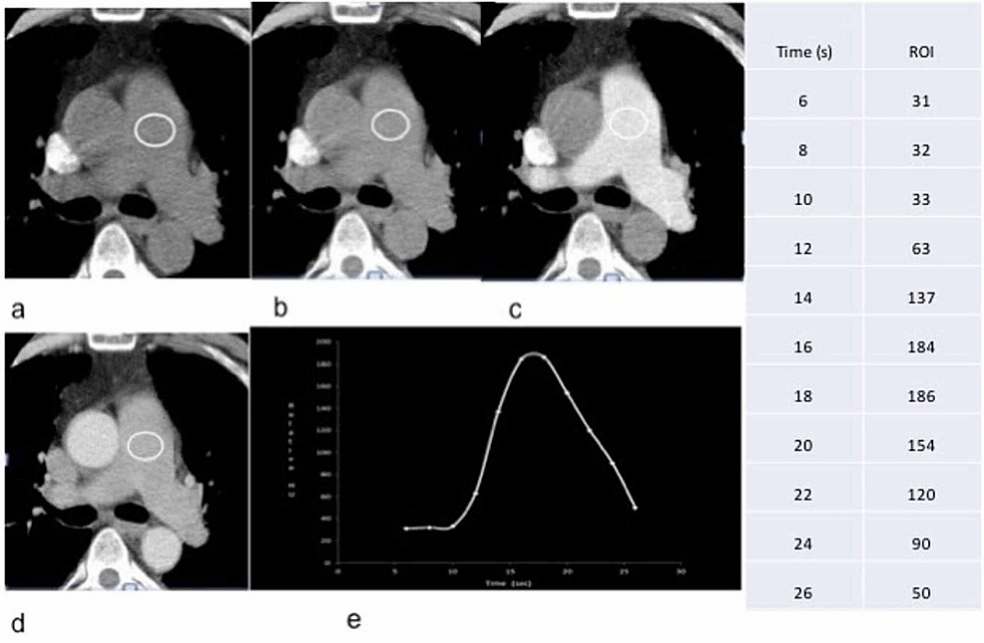
Time bolus curve Representative images from a test bolus study (a to d) showing region of interest (ROI) within the center of main pulmonary artery and dynamic enhancement of the artery. Measurements obtained at times shown on the right hand side of the image. A time attenuation curve was generated (e). Measured full width at half maximum (FWHM) from this curve was 9. On this patient mean pulmonary artery pressure (mPAP), pulmonary vascular resistance (PVR) and indexed PVR (PVRI) on right heart catheterization performed within one week were 45 mmHg, 7.4 Wood units and 4.3 correspondingly.

**Figure 2:**
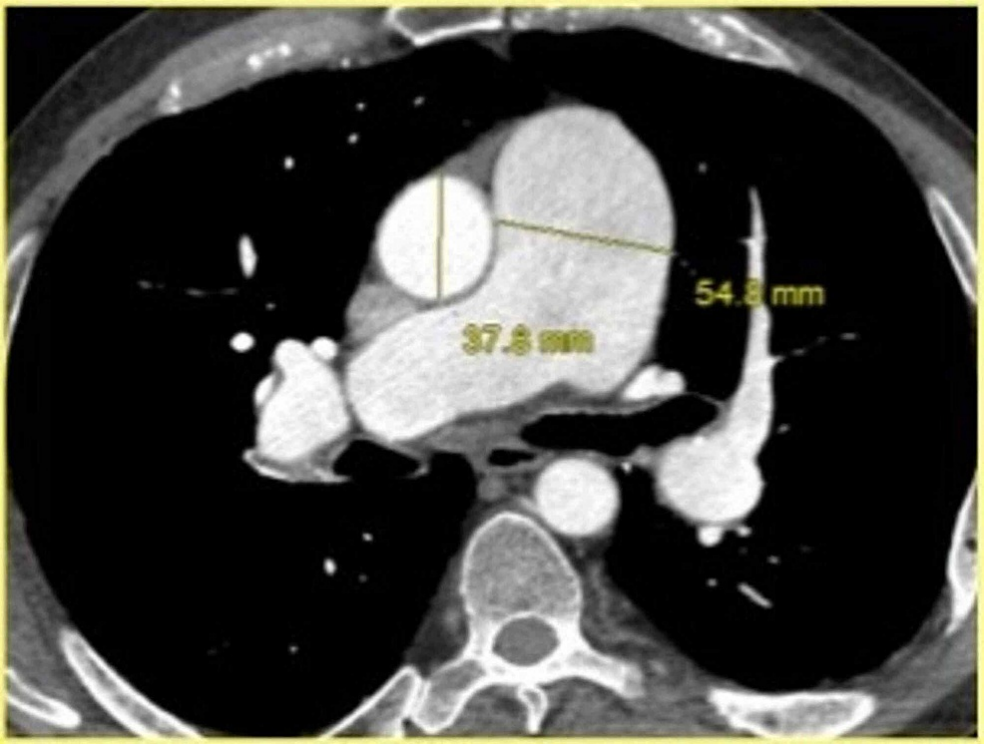
Example of measurement of main pulmonary artery (MPA) diameter in a patient with pulmonary hypertension. Diameter of the MPA on cross-sectional imaging was measured in the scan plane of the pulmonary artery bifurcation where right main pulmonary artery is visualized in its extent, at a right angle to its long axis. On this patient, the diameter was 5.5 cm. MPA/aorta diameter ratio was 1.4.

Figure 1 shows an example of FWHM measurement in a patient with known pulmonary hypertension. The conventional parameters were measured on the axial images of the diagnostic scans. Diameter of the MPA on cross-sectional imaging was measured at a right angle to its long axis in the scan plane of the pulmonary artery bifurcation where the right main pulmonary artery is visualized in its extent (Figure 2).

Similarly, aortic diameter was obtained at the same level for assessment of MPA/aorta ratio (Figure 2). Additional parameters that were evaluated included right/left ventricular ratio (RV/LV), interventricular septal position and diameters of the right and left main pulmonary arteries. RHC data were obtained from electronic medical record (EMR) and included mPAP, PVR and indexed PVR (PVRI). An mPAP of more than and/or equal to 25 mm Hg at rest was considered significant in a patient with pulmonary hypertension [1,2]. A PVR value of more than 3 Woods units was also considered abnormal [1,2,8,9].

Statistical analysis

Pearson correlation coefficients were calculated by using Microsoft Excel software between FWHM with mPAP, PVR and PVRI. Correlations with these angiographic parameters were also assessed with all other conventional parameters measured from the CT test bolus data. A P-value of <0.05 was considered statistically significant.

## Results

Patient population and demographics

We identified 221 patients in our database who had undergone CTPA study between January 2010 to December 2013 for evaluation of pulmonary hypertension. Among those patients, 52 were identified who had a CTPA study for evaluation of pulmonary hypertension and also had an RHC within one month of CTPA. Of these 52 patients, 37 fulfilled the selection criteria. Eight patients were excluded due to breathing motion and streak artifacts during contrast bolus. Seven patients were excluded due to inadequate duration of test bolus injection. Our selected patient population comprised 26 female and 11 male patients with an age range between 30 to 85 years.

Correlation of test bolus (CTPA) with right heart catheterization data

**Figure 3:**
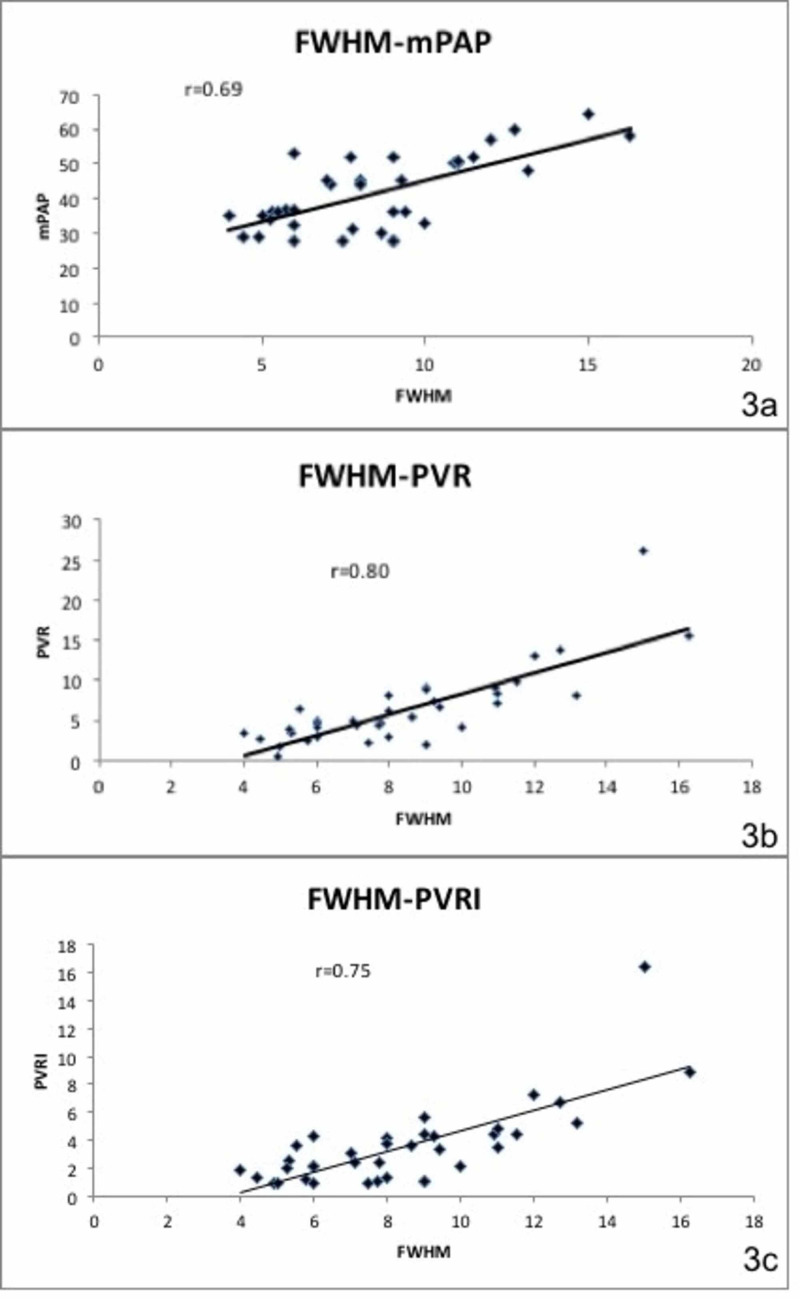
Full width at half maximum (FWHM) and right heart catheterization (RHC) parameters. Correlation of FWHM with RHC parameters, including mean pulmonary artery pressure (mPAP; 3a), pulmonary vascular resistance (PVR; 3b) and indexed PVR (PVRI; 3c).

A correlation of established CT parameters including main pulmonary artery diameter (MPA), right and left main pulmonary artery diameters (RPA and LPA), MPA/aorta ratio, and RV/LV ratio was obtained with RHC data (Table 1, Figure 3). Table 1 also shows correlation of all CTPA parameters with right heart catheterization data. 

**Table 1:** Correlation of Computed Tomography pulmonary angiography (CTPA) parameters with right heart catheterization parameters. FWHM – full width at half maximum, MPA – main pulmonary artery, RPA – right pulmonary artery, LPA – left pulmonary artery, RV/LV – right and left ventricular ratio, A/B – arterial and bronchial ratio, MPA/Ao – MPA and aorta ratio, mPAP – mean pulmonary artery pressure, PVR – Pulmonary Vascular Resistance, PVRI – indexed PVR.

Parameters	Correlation coefficient/ P- value					
	mPAP		PVR		PVRI	
FWHM	0.69	<0.00001	0.80	<0.00001	0.75	<0.00001
MPA	0.26	0.1135	0.05	0.7783	0.01	0.9468
RPA	0.24	0.1582	0.03	0.8707	0.08	0.6584
LPA	0.15	0.3854	0.11	0.5348	0.15	0.3837
RV/LV	0.03	0.8445	0.16	0.344	0.15	0.39
MPA/Ao	0.01	0.9646	0.02	0.8855	0.07	0.6901

There was a strong correlation between FWHM and mPAP (r=0.69, p value<0.00001), PVR (r=0.8, p value<0.00001) and PVR (r=0.75, p value<0.00001) (Figure 3a, 3b, 3c). Whereas the correlation between MPA, RPA, LPA, MPA/aorta ratio, arterial and bronchial ratio (A/B) and RV/LV and mPAP, PVR and PVRI was poor (0.1-0.32).

## Discussion

RHC hemodynamic measurements, including mPAP, PVR and PVRI, are markers of severity of pulmonary hypertension. They are also used to predict prognosis and survival in patients with PAH [10]. In this retrospective study, we evaluated the correlation between various conventional and new MPA contrast kinetic measurements from CTPA with RHC metrics. 

Increased mPAP and PVR lead to eventual right ventricle (RV) dysfunction [11]. Changes in pressure and resistance in the pulmonary circulation lead to changes in contrast flow kinetics. With elevated pressure and resistance, there is slow transit through the pulmonary circulation that is reflected as increased FWHM. Studies performed with contrast enhance MRI to evaluate contrast flow kinetics have shown good correlation of FWHM with RHC measurements [3], good correlations of pulmonary artery to pulmonary vein transit time with systolic and mean PAP [12] and significantly increased flow parameters (including mean transit time and time to peak) with elevated PAP and PVRI [13]. Similar results are seen in the present study using CTPA test bolus data. To our knowledge, correlation of these angiographic parameters was not performed till now with easily available CTPA test bolus kinetics information. 

Dilatation of the central pulmonary arteries can be seen in patients with PAH. Previous studies have shown that a diameter of MPA measuring 29 mm or more has 87% sensitivity and 89% specificity for PAH [6]. However, in our analysis, the diameters of central pulmonary arteries (including MPA, LPA and RPA) didn’t show a significant correlation with right heart catheterization parameters for pulmonary hypertension. Ng et al. have shown that a ratio of MPA/aorta diameter of more than one has a good correlation with elevated mean pulmonary artery pressure [14]. In our study, the correlation was not significant when compared to hemodynamic parameters including mPAP, PVR and PVRI. Dilatation of RV is described in patients with pulmonary hypertension of any cause. Relative enlargement of the RV as compared to the left ventricle (LV) with an RV/LV ratio of more than one correlates with adverse clinical events [15]. However, there was no significant correlation of RV/LV ratio with RHC markers in our study. Prior studies that focused on the conventional measurements were done as comparison between patients with and without PAH, showing differences between the two groups. However, in the current study, we are comparing individual physiological parameters measured from RHC in established cases of PAH to evaluate their predictive power. Further, the conventional parameters are focused on morphologic or structural changes that may be time-dependent and multifactorial, whereas the temporal change in the CT attenuation data is a true reflection of physiological alterations in the pulmonary circulation and thus shows a stronger correlation.

We recognize a few limitations of our study. This is a retrospective study with a small sample size. Patient factors other than altered pulmonary hemodynamics can also affect the density measurements, such as variation in the phase of respiration, dilution caused by unopacified blood pool from inferior vena cava, differences in end diastolic right ventricular volume, pulmonary valvular regurgitation, and heart rate variation to name a few. Anatomic factors such as site of contrast injection (e.g. antecubital vein vs. a smaller vein over the wrist) and body habitus were not controlled. Our study lacked a control group without pulmonary hypertension. Another limitation is the rate of contrast injection may not be similar in different institutions, which may change the graph. We think that further research with a larger cohort, inclusion of normotensive patients and assessment of subtypes of pulmonary hypertension will provide a better perspective.

## Conclusions

FWHM calculated from the dynamic images during test bolus of CTA is an easily available, simple to perform, reliable, and noninvasive quantitative measure. Our study shows a very strong correlation between this CTA parameter and mPAP and PVR/PVRI obtained on right heart catheterization in pulmonary hypertension, whereas the traditionally used morphological CT measurements such as diameter of main and branch pulmonary arteries or RV/LV ratio did not show significant correlation.

Since CTPA is performed as a part of routine workup for pulmonary hypertension, this has a potential for diagnosis as well as follow-up for assessing the treatment response or disease progression. It may also help to detect subclinical cases of pulmonary hypertension on CTPA studies done for other indications enabling early diagnosis and treatment.
